# Incidence Trends of Primary Cutaneous T-Cell Lymphoma in the US From 2000 to 2018: A SEER Population Data Analysis

**DOI:** 10.1001/jamaoncol.2022.3236

**Published:** 2022-09-01

**Authors:** Zhuo Ran Cai, Michael L. Chen, Martin A. Weinstock, Youn H. Kim, Roberto A. Novoa, Eleni Linos

**Affiliations:** 1 Program for Clinical Research and Technology, Stanford University, Stanford, California; 2 Department of Dermatology, Stanford University School of Medicine, Stanford, California; 3 Center for Dermatoepidemiology, Providence Veterans Affairs Medical Center, Providence, Rhode Island; 4 Department of Dermatology, Brown University, Providence, Rhode Island; 5 Department of Epidemiology, Brown University, Providence, Rhode Island; 6 Department of Medicine (Oncology), Stanford University School of Medicine, Stanford, California; 7 Department of Pathology, Stanford University School of Medicine, Stanford, California; 8 Stanford Cancer Institute, School of Medicine, Stanford, California

## Abstract

Using SEER database data, this cohort study analyzed cutaneous T-cell lymphoma incidence by tumor subtype, sex, age, race and ethnicity, socioeconomic status, and geography.

Recent incidence patterns of cutaneous T-cell lymphoma (CTCL) in the US are not well described. We sought to describe recent incidence trends by tumor subtype, sex, age, race and ethnicity, socioeconomic status (SES), and geography.

## Methods

Incidence data were derived from 18 population-based registries of the Surveillance, Epidemiology, and End Results (SEER) Program for 2000 to 2018. We included histologically confirmed cases of first primary CTCL with malignant behavior and primary site of involvement skin (C44.0-C44.9). Patient characteristics included sex, age, race and ethnicity, and geographic region (metropolitan vs nonmetropolitan counties).^1^ Area-level SES information was available from 2000 to 2016 and was categorized into quintile; Q1 lowest, Q5 highest.^2^ We used the 2000 US standard population to calculate age-adjusted annual incidence rates (IR) per million people. Annual percent change (APC) was calculated by using the weighted least squares method. Statistical calculations used SEER*Stat statistical software (version 8.3.9.2) in January 2022. The Stanford institutional review board deemed this study exempt from review and waived the requirement for patient informed consent because only deidentified data were used.

## Results

We identified 14 942 new cases of CTCL from 2000 to 2018. Table 1 shows the number of cases, IR, and APC of IR of CTCL by *International Statistical Classification of Diseases and Related Health Problems for Oncology (ICD-O)* diagnosis. Mycosis fungoides (MF) was the most common diagnosis, followed by primary CTCL (PCTCL) and primary cutaneous anaplastic large cell lymphoma (PCALCL). The overall CTCL incidence was 8.55 per million and increased over the study period (APC, 0.61%). Among CTCL subtypes, MF had the highest incidence (5.42) and Sézary syndrome had the highest increase (APC, 3.83%). Overall, PCTCL was the only subtype with decreasing incidence over the study period (APC, −1.39%).

**Table 1.** Number of Cases, Age-Adjusted Incidence Rate, and Annual Percent Change of Incidence Rate of CTCL in 18 SEER Registries Between 2000 and 2018

Subtypes (*ICD-O*)	Cases, no.	Frequency, %	Incidence rate (per million people)	Annual % change (95% CI)
All CTCL	14 942	100	8.55	0.61 (0.12 to 1.10)^a^
Mycosis fungoides (9700/3)	8445	56.6	5.42	1.34 (0.89 to 1.79)^a^
Sézary syndrome (9701/3)	268	1.8	0.21	3.83 (2.00 to 5.69)^a^
Peripheral T-cell lymphoma, not other specified (9702/3)	910	6.1	0.48	0.31 (−0.93 to 1.56)
Subcutaneous panniculitis-like T-cell lymphoma (9708/3)	61	0.4	0.02	NA
Primary CTCL (9709/3)	3637	24.3	1.50	−1.39 (−2.22 to −0.56)^a^
Primary cutaneous anaplastic large cell lymphoma (9718/3)	1509	10.1	0.86	−0.48 (−2.57 to 1.65)
Extranodal natural killer/T-cell lymphoma (9719/3)	52	0.3	0.01	NA
Primary cutaneous γ-δ T-cell lymphoma (9726/3)	48	0.3	0.05	NA
Adult T-cell leukemia/lymphoma (9827/3)	12	0.1	0.01	NA

Abbreviations: CTCL, cutaneous T-cell lymphoma; *ICD-O*, *International Classification of Diseases for Oncology*; NA, not applicable; SEER, Surveillance, Epidemiology, and End Results.

^a^ Significantly different from 0.

Table 2 shows IR and APC by tumor subtype, sex, age, race and ethnicity, SES, and geographic region. Overall, CTCL incidence was highest in men (10.06), non-Hispanic Black patients (11.68), individuals in highest SES quintiles (10.31), and patients living in metropolitan counties (8.96). Patients aged 40 years or older had a 6-times higher overall incidence rate than those younger than 40 years. Despite having a lower incidence compared with the older age group, patients younger than 40 years showed significantly higher increases in overall CTCL IR (APC, 2.87%) and in MF IR (APC, 3.67%). Other groups with significant increases in incidence included women (APC, 0.92%), non-Hispanic Black patients (APC, 1.63%), patients in the lowest SES quintile (APC, 1.87%), and individuals in metropolitan counties (APC, 0.68%).

**Table 2.** Incidence Rate in 2018 and Annual Percent Change of CTCL, Mycosis Fungoides, Sézary Syndrome, and Primary Cutaneous Anaplastic Large-Cell Lymphoma by Sex, Age, Race and Ethnicity, SES, and Geographic Location

Characteristics	All CTCL		Mycosis fungoides		Sézary syndrome		Primary cutaneous anaplastic large cell lymphoma	
	Rate	APC, % (95% CIs)	Rate	APC, % (95% CIs)	Rate	APC, % (95% CIs)	Rate	APC, % (95% CIs)
Sex								
Men	10.06	0.21 (−0.36 to 0.79)	6.23	1.08 (0.43 to 1.73)^a^	0.24	2.47 (0.20 to 4.78)^a^	1.05	−0.82 (−3.19 to 1.61)
Women	7.32	0.92 (0.34 to 1.50)^a^	4.78	1.48 (0.82 to 2.14)^a^	0.18	4.99 (2.17 to 7.89)^a^	0.70	−0.52 (−2.90 to 1.92)
Age group, y								
<40	2.52	2.87 (1.57 to 4.19)^a^	1.79	3.67 (2.42 to 4.93)^a^	0.02	NA	0.17	−0.49 (−4.68 to 3.88)
≥40	16.52	0.19 (−0.26 to 0.66)	10.22	0.83 (0.37 to 1.30)^a^	0.45	3.28 (1.45 to 5.13)^a^	1.77	−0.45 (−2.47 to 1.61)
Race and ethnicity								
Black	11.68	1.63 (0.80 to 2.47)^a^	6.50	2.03 (0.85 to 3.23)^a^	0.45	NA	1.64	1.38 (−2.57 to 5.50)
Hispanic	7.07	1.12 (−0.01 to 2.26)	4.94	1.85 (0.39 to 3.33)^a^	0.20	NA	0.60	−0.35 (−3.85 to 3.26)
Non-Hispanic								
White	7.89	0.14 (−0.39 to 0.68)	4.92	0.88 (0.39 to 1.36)^a^	0.17	2.41 (0.75 to 4.10)^a^	0.80	−0.71 (−2.77 to 1.39)
SES (quintiles)^b^								
Q1 (lowest)	7.55	1.87 (0.53 to 3.23)^a^	4.51	1.69 (0.13 to 3.26)^a^	0.15	NA	0.78	2.91 (−2.09 to 8.16)
Q2	7.40	1.34 (0.37 to 2.32)^a^	4.26	2.12 (0.74 to 3.52)^a^	0.12	0.70 (−3.89 to 5.51)	0.94	−0.05 (−3.64 to 3.68)
Q3	7.47	0.92 (−0.15 to 2.01)	4.10	1.33 (−0.10 to 2.78)	0.38	NA	0.90	NA
Q4	8.91	0.52 (−0.79 to 1.85)	4.98	0.75 (−0.76 to 2.29)	0.05	NA	1.10	−0.19 (−3.07 to 2.77)
Q5 (highest)	10.31	1.16 (0.27 to 2.06)^a^	6.99	1.96 (0.93 to 3.00)^a^	0.15	NA	0.66	0.00 (−2.98 to 3.06)
Geographic location								
Metropolitan	8.96	0.68 (0.16 to 1.20)^a^	5.76	1.38 (0.85 to 1.92)^a^	0.20	4.26 (2.11 to 6.46)^a^	0.86	−0.47 (−2.50 to 1.60)
Nonmetropolitan	4.85	−0.66 (−2.03 to 0.73)	2.40	−0.19 (−2.09 to 1.75)	0.26	NA	0.76	−0.80 (−4.31 to 2.85)

Abbreviations: APC, annual percent change; CTCL, cutaneous T-cell lymphoma; NA, not applicable; SES, socioeconomic status.

^a^ Significantly different from 0.

^b^ Data limited to patients diagnosed from 2000 to 2016.

## Discussion

We observed an increased overall CTCL incidence between 2000 and 2018. These findings differ from previous North American studies,^3,4^ which suggested CTCL incidence stabilized after 1998, but are consistent with recent studies from Europe.^5^ These trends are likely multifactorial. Better diagnostic tools and increased awareness among physicians and patients may have led to improved CTCL detection. Physician density has been associated with higher incidence^6^; therefore, efforts to increase access to health care may contribute to a rise in CTCL diagnosis. The incidence of PCTCL has decreased considerably since 2000, possibly owing to better classification because PCTCL is often a diagnosis of exclusion.

Overall, CTCL incidence by age, sex, and race and ethnicity stayed mostly consistent with previous reports.^1,6^ One key new finding is the more rapid increase in incidence among young patients, possibly owing to earlier diagnosis. Another hypothesis is that MF cases previously misdiagnosed as atopic dermatitis or psoriasis are discovered at a younger age in the era of biologics. Higher SES and metropolitan county were both associated with increased incidence, consistent with previous studies^6^ correlating with income and physician density. Environmental exposures may also be associated with CTCL owing to clustering of cases in industrial regions of North American cities.^4^

To our knowledge, this is the first study to analyze CTCL incidence by SES and geography. Limitations include potential underreporting, heterogeneous classification, changes in diagnostic tools, and small sample sizes.

These findings suggest that the incidence of CTCL continues to increase in the US. Prospective data collection efforts should gather data on SES, geographic location, and health care access to better understand these differences.
